# Cine and tagged cardiovascular magnetic resonance imaging in normal rat at 1.5 T: a rest and stress study

**DOI:** 10.1186/1532-429X-10-48

**Published:** 2008-11-03

**Authors:** Jean-Luc Daire, Jean-Pascal Jacob, Jean-Noel Hyacinthe, Pierre Croisille, Karin Montet-Abou, Sophie Richter, Diomidis Botsikas, Matthieu Lepetit-Coiffé, Denis Morel, Jean-Paul Vallée

**Affiliations:** 1 Radiology Department, Faculty of Medicine, University of Geneva and Geneva University Hospital, CH-1211 Geneva 14, Switzerland; 2 Radiology Department, Hopital L. Pradel, CREATIS UMR CNRS 5515 & INSERM U630 UCB, Lyon I, France; 3 Anesthesiology, Pharmacology and Intensive Care Department, Faculty of Medicine, University of Geneva and Geneva University Hospitals, CH-1211, Geneva 14, Switzerland

## Abstract

**Background:**

The purpose of this study was to measure regional contractile function in the normal rat using cardiac cine and tagged cardiovascular magnetic resonance (CMR) during incremental low doses of dobutamine and at rest.

**Methods:**

Five rats were investigated for invasive left ventricle pressure measurements and five additional rats were imaged on a clinical 1.5 T MR system using a cine sequence (11–20 phases per cycle, 0.28/0.28/2 mm) and a C-SPAMM tag sequence (18–25 phases per cycle, 0.63/1.79/3 mm, tag spacing 1.25 mm). For each slice, wall thickening (WT) and circumferential strains (CS) were calculated at rest and at stress (2.5, 5 and 10 μg/min/kg of dobutamine).

**Results:**

Good cine and tagged images were obtained in all the rats even at higher heart rate (300–440 bpm). Ejection fraction and left ventricular (LV) end-systolic volume showed significant changes after each dobutamine perfusion dose (p < 0.001). Tagged CMR had the capacity to resolve the CS transmural gradient and showed a significant increase of both WT and CS at stress compared to rest. Intra and interobserver study showed less variability for the tagged technique. In rats in which a LV catheter was placed, dobutamine produced a significant increase of heart rate, LV dP/dtmax and LV pressure significantly already at the lowest infusion dose.

**Conclusion:**

Robust cardiac cine and tagging CMR measurements can be obtained in the rat under incremental dobutamine stress using a clinical 1.5 T MR scanner.

## Background

The clinical hallmarks of hibernating myocardium in chronic ischemic patients include regional contraction deficit while retaining an inotropic reserve during a low dose dobutamine challenge. Experimental models on rodents aiming to reproduce myocardial hibernation require an accurate quantification of the contractile reserve measured after low dose of dobutamine. Currently, two modalities echocardiography [1] and cardiovascular magnetic resonance (CMR) can provide cardiac function measurement in rodents. While echocardiography has been recently used to study cardiac response under dobutamine in normal rat [1], it is not routinely performed in rodent experiments. Echocardiography remains operator dependant and its reproducibility still needs to be largely validated. By contrast, CMR is operator independent and is generating a growing interest for rodents cardiac function analysis in the research community [2,3]. Cardiac function using either cine or tagged CMR have been successfully and reproducibly obtained in rodents.

Cine CMR consists in the acquisition of the same slice position at different phases of the cardiac cycle. It allows the assessment of left ventricle (LV) size and shape as well as global LV function and wall thickening [4-6]. In normal and infarcted mice, cine CMR was performed at rest and after administration of 1.5 μg/g intraperitoneal dobutamine [7]. Heart rate, cardiac output, and ejection fraction were significantly increased whereas end-diastolic and end-systolic left ventricular (LV) volumes were decreased in normal mice during dobutamine challenge. In mice with heart failure due to chronic myocardial infarction (MI), dobutamine did not increase LV dynamics reflecting an absence of contractile and relaxation reserve.

Intra-myocardial mechanisms including wall motion strains and/or torsion angle, can be measured when presaturation tag pattern (CMR-tagging) is added to cine imaging. The basic principle is to tag the myocardium physically using spatially selective saturation pulses and to track the displacement of the tagged myocardium. CMR-tagging has been well demonstrated in normal rats [8-10]. Myocardial function after infarct has been studied by tagged CMR [11-13] and a decrease in the transmural gradient of circumferential strain (CS) has been demonstrated in rats [12]. However, no previous study has described the incremental contractile response to graded doses of dobutamine using tagged CMR in normal rats. CMR-tagging has been used successfully with dobutamine in a single previous report to evaluate cardiac function in neuronal nitric oxide synthase knockout mice [13]. Wild type mice were used as control, but no value of the dobutamine induced transmural variations of the circumferential strains as well as no reproducibility study were reported. In addition only high doses of dobutamine (20 or 40 μg/min/kg) were used.

The purpose of the present study was to evaluate the effects of low dose dobutamine on the myocardium contraction in rat using tagged and cine CMR on a 1.5 T clinical system.

## Materials and methods

### Animal preparation

Ten normal adult *Sprague-Dawley *rats (weighing 385 ± 30 g) were included in the protocol approved by the ethical committee of our institution. Five rats were used for MR imaging, and five additional rats were investigated for invasive LV pressure measurements.

### CMR

All images were acquired on a clinical scanner at 1.5 T with a microscopic surface coil (47 mm diameter) (Intera, Philips Medical System, Best, NL). Anesthesia was induced and maintained with isoflurane 3% administered by a facemask, with animals breathing spontaneously oxygen enriched air (FIO_2_: 0.4). The rats were placed in a cradle in prone position. A rectal thermal probe with a heating system maintained the rat's temperature constant (37.0–37.5°C). A respiratory pillow was placed under the abdomen to monitor respiratory rate. Subdermal electrodes recorded an electrocardiogram (ECG) that was used to trigger the MR system. The monitoring devices were connected with optical fibers to a computer during the MR acquisitions (SA Instrument, Inc (SAII), Stony Brook, NY, MR-Compatible Model 1025 Monitoring and Gating System). Using this set-up, the physiological parameters of the rats were continuously measured in real time during the whole experiment.

Cine CMR was realized using a prospective ECG-triggered segmented turbo field echo cine sequence, TR/TE 12/4.9 ms. Flip angle (FA) 30°, 288 × 288 matrix sampled on a Cartesian grid, 80 mm FOV and 2 mm slice thickness yielding an acquired voxel resolution of 0.28 × 0.28 × 2 mm. Eleven to twenty cardiac phases were acquired per R-R cycle depending on the heart rate. 2 short axis levels and two- and four-chambers were realized at rest and for each dobutamine dose.

Tagged CMR was obtained by using a C-SPAMM tag preparation segmented cine fast field echo sequence, TR/TE 7.8/3.6 ms, tag spacing 1.25 mm, acquired voxel size 0.63 × 1.79 × 3 mm, FA 10°. The inter image was about 8 msec yielding to 18–25 phases per cardiac cycle. 2 short axis levels were realized at rest and for each dobutamine dose

### Stress study

Dobutamine 0.5% (Fresenius Kabi AG, Stans, Switzerland) diluted to a final concentration of 50 μg/ml with isotonic saline was consecutively administered for 20 min as a continuous intravenous infusion at 2.5, 5 and 10 μg/min/kg rate of injection by means of a continuous infusion pump (Welmed P100, Arcomed AG Medical Systems, Regensdorf, Switzerland): CMR image acquisition started once the heart rate was stabilized (between 5 and 10 min after start of drug infusion).

### Hemodynamic study

Animals were conditioned similarly as for imaging and anesthetized with isoflurane by facemask, intubated and mechanically normo-ventilated with oxygen-enriched air (FIO_2_, 0.4) at a tidal volume of 7 ml/kg body weight and a respiratory rate of 70 bpm with a constant volume-cycled rodent ventilator (model 683, Harvard Apparatus Co Inc., South Natick, MA). A positive end-expiratory pressure of 2.5 cm H_2_O was applied. Anesthesia was maintained with 1.4% isoflurane. Respiratory gases and airway pressure were continuously monitored. A 3-lead ECG was recorded on a Hewlett-Packard monitor using subcutaneous electrodes. The femoral vein was cannulated for drug infusion and the carotid artery for continuous arterial blood pressure monitoring using a calibrated pressure transducer (Honeywell, model 156 PC 06-GW2, Zürich, Switzerland). Through a left thoracotomy, the heart was exposed and a 20-cm long polyethylene catheter (ID 0.58 mm, OD 0.96 mm) connected to a second calibrated pressure transducer was placed into the LV through the apex for continuous LV pressure measurement. The transducers were further connected to a pressurized continuous normosaline filled flushing system (250 mmHg; IntraFlo^®^). Meticulous care was taken to ensure that the catheters and pressure transducer systems remained free of air bubbles during the experimental protocol. The thorax was then closed.

For all rats, after several sighs to remove residual pleural air, anesthesia was lightened to allow spontaneous breathing through the endotracheal cannula with its gas and pressure sampling port left in place. The measured hemodynamic and respiratory variables were continuously recorded and stored at a sampling rate of 1000 Hz via an analog/digital interface converter (Biopac, Santa Barbara, CA, USA) on an Acer microcomputer for further off-line analysis. Rectal temperature was maintained constant with a heating pad.

After a stabilization period of 30 min under stable isoflurane anesthesia (0.85%) with animals breathing spontaneously oxygen enriched air (FIO_2_: 0.4) and documentation of normal pH and PaCO_2 _concentration by arterial blood gas analysis, the stress study was started. Systolic, diastolic and mean arterial pressure, heart rate, LV systolic and end-diastolic pressures, the maximal rate of pressure development (LV dP/dtmax), and peak LV pressure were measured during baseline conditions and during a dobutamine challenge identical to the protocol used during the MR sessions.

### Cardiac function analysis

Image analysis was performed using the open source software OsiriX . Left ventricle ejection fraction (LVEF) was assessed for each animal with MR cine images according to: LVEF(%) = (V_diast _- V_syst_)/V_diast_. End-diastolic (V_diast_) and end-systolic (V_syst_) volumes were calculated by manually drawing endocardial contours and long axis length at end-diastolic and end-systolic phases for each axis. End-diastolic and end-systolic volumes were calculated according to the Simpson's modified method [14]: Volume = (length/2) × (A_basal_+2/3 × A_apex_) with A_basal_, diastolic or systolic basal area; A_apex_, diastolic or systolic area and length, diastolic or systolic long axis length.

### Myocardium wall thickening

Regional contraction analysis was performed by measuring myocardium thickness changes between diastolic and systolic phases on cine images. Endocardium and epicardium boundaries were manually segmented on end-diastolic and end-systolic phase images. The myocardial slices were then divided in 128 evenly spaced radial sectors and then averaged into six main sectors. The wall thickening was obtained by subtraction of diastolic and systolic radii lengths.

### Tag analysis

The tag analysis was performed with the Extrema Temporal Chaining (ETC) algorithm[15]. ETC algorithm consists in tracking points of tag and crest lines along their orthogonal direction by a temporal chaining. These points are detected as local minima and maxima of 1D signals corresponding to lines or columns of the 2D images. In parallel, a spatial smoothness assumption of the deformation field and to the myocardial boundaries is incorporated to prevent abnormal extrema matching. Starting from a spatial chaining on the first image, real 2D displacements are computed on extrema locations. Finally, displacements of all the myocardium points are interpolated via the Laplace equation. Circumferential strains (CS) are then averaged in six sectors. Maximum of the CS was measured in % according to the formula: %CS = (CS_syst _- CS_dias_). We defined CSepi, CSmid and CSendo at epicardial, midwall and endocardial levels respectively by dividing the wall thickness in thirds.

Intra and inter observer study: three observers were blinded of dobutamine administration and pathological status of rats. Two rats were analyzed in this way including 2 short axis levels per animal, at rest and under stress (3 doses), yielding 16 slices per sequence. Each observer performed three measurements with at least a one week interval between each measurement.

Value per sector of the three observers or different measurement were averaged and the ratio of the standard deviation by this mean was obtained for each sector to compare the intra and inter observer variability percentage in cine and tagged images.

### Statistical analysis

All data are presented as means ± standard deviation. Data from the invasive hemodynamic studies were averaged over the last 10 heart beats before switching to the next dobutamine dosage. The difference between doses groups and modalities were determined by using an one-way ANOVA with repeated measures design followed by post hoc Newman-Keuls multiple comparison test. All data are expressed as means ± standard deviation. A p value less than 0.05 was considered statistically significant.

## Results

**Figure 1:**
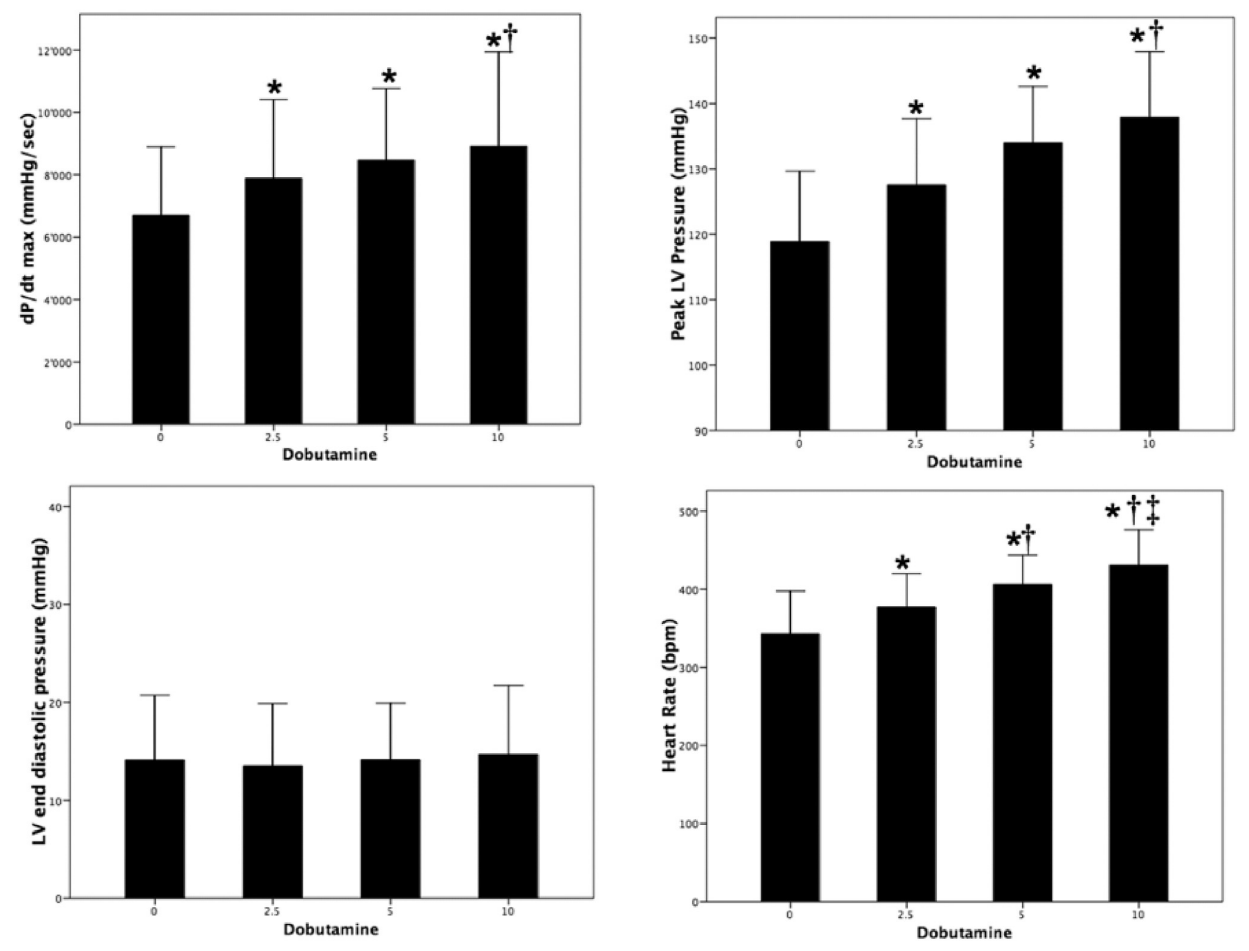
Haemodynamic response to an incremental stress using incremental dobutamine doses: 0, 2.5, 5 and 10 μg/min/kg (*: p < 0.05 vs 0; †: p < 0.05 vs 2.5; ‡:p < 0.05 vs 5 μg/min/kg).

In rats in which a LV catheter was placed, dobutamine produced the well known expected chronotropic and inotropic effects (fig. 1). Both heart rate and LV dP/dtmax increased significantly (p < 0.01) already at the lowest infusion dose (+ 11% and + 18%, respectively), and then further increased with the larger dobutamine dosages (+ 27% and + 34% at 10 μg/min/kg). Although peak LV pressure increased with incremental dobutamine doses, this effect was not associated with a significant raise in mean systemic arterial pressure. In addition, dobutamine infusion did not change LV end-diastolic pressure, ECG shape (ST-segment elevation), or respiratory rate (60 – 65 bpm).

**Figure 2:**
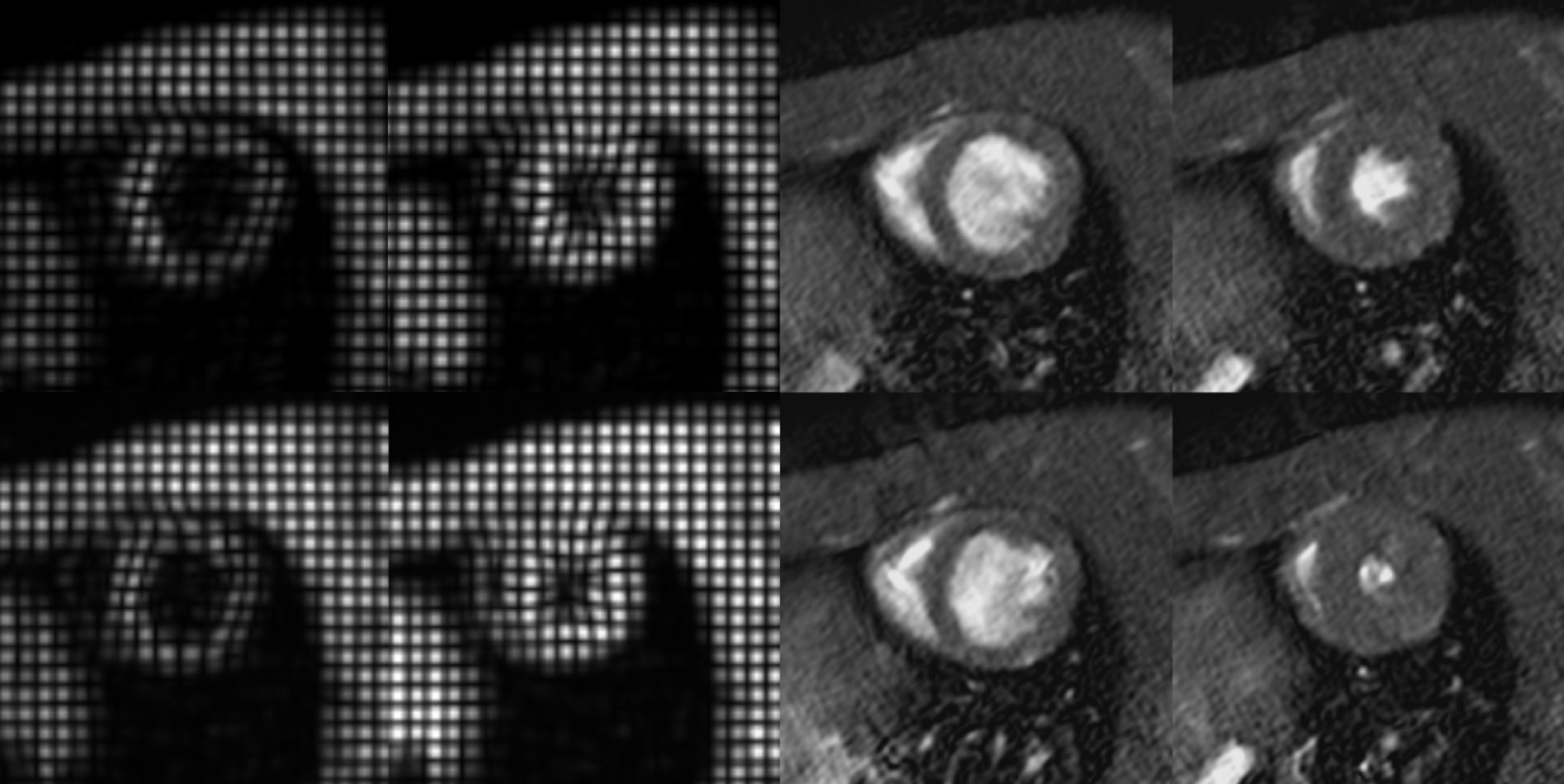
**Representative example of tagged and cine CMR at rest (first row) and stress under 5 μg/min/kg of dobutamine (2^nd ^row) in diastolic and systolic phases obtained in normal heart. **The effect of both the increase of wall thickening and the decrease of the LV end-systolic volume are clearly seen on the CMR images.

Regarding the imaging study, high image quality of both cine and tagged CMR was obtained in all the rats at rest and after dobutamine as shown in figure 2. Due to the high spatial resolution and the absence of significant flow artifact, anterior and posterior papillary muscles were clearly identified on cine CMR during the complete dobutamine challenge. Since 11 to 20 cardiac phases were achievable with cine CMR, there was no ambiguity to define the diastolic and systolic phases for the quantitative analysis even at high heart rate (350–450 bpm) observed during the 10 μg/min/kg dobutamine infusions. Even at high heart rate in rats, tag fading was virtually absent yielding to enhanced contrast between the tag lines as well as complete coverage of the cardiac cycle. A transmural CS gradient was measured in all the rats at rest (CSendo/CSepi = 23.0% ± 6.9%).

**Figure 3:**
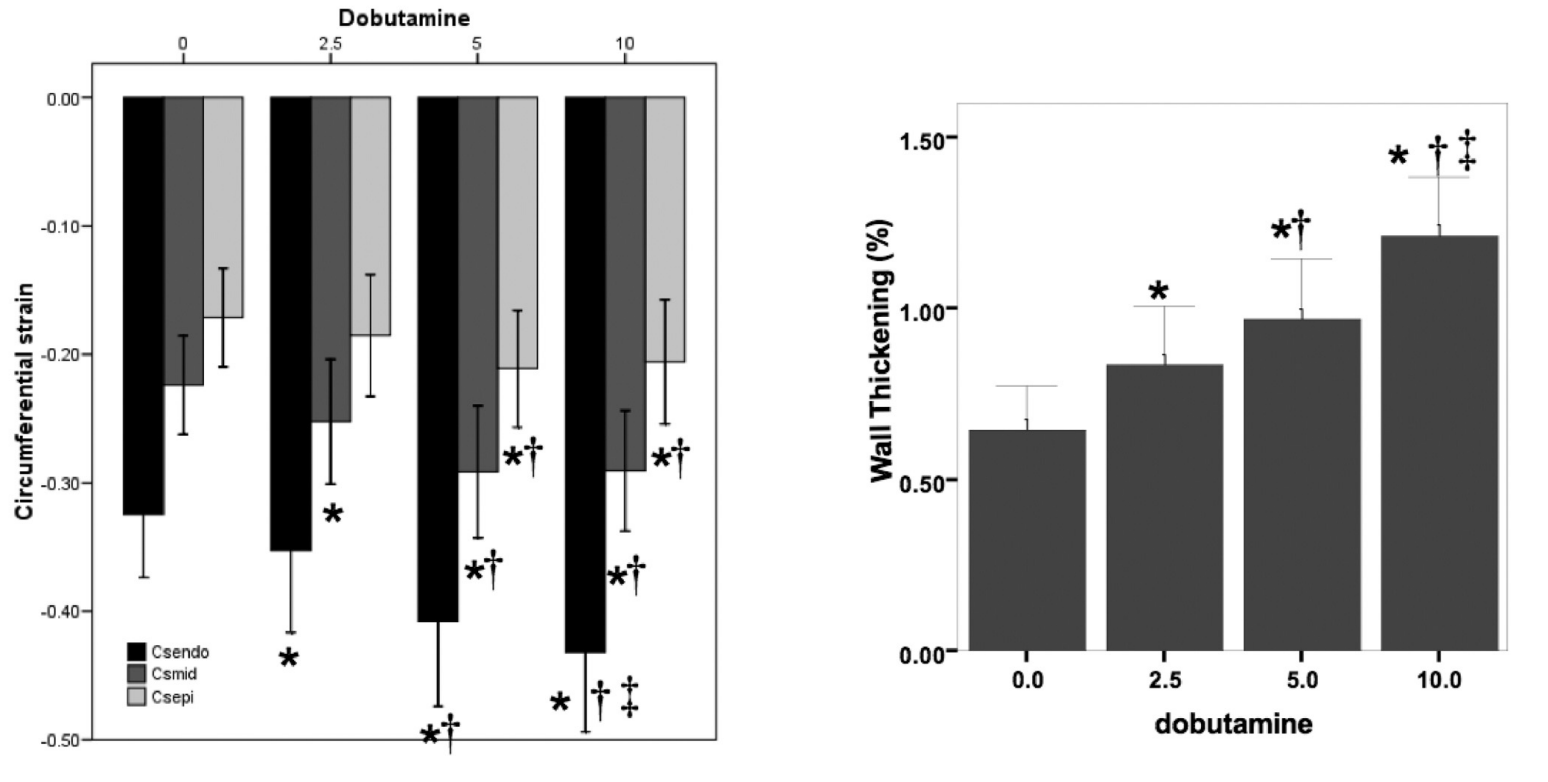
**Wall thickening and circumferential strain at endocardial, midwall and epicardial levels measurements.** All measurements under dobutamine stress are significantly different from the values obtained at rest, except for circumferential strain at epicardial at 2.5 μg/min/kg. (*: p < 0.05 vs 0; †: p < 0.05 vs 2.5; ‡:p < 0.05 vs 5 μg/min/kg).

**Figure 4:**
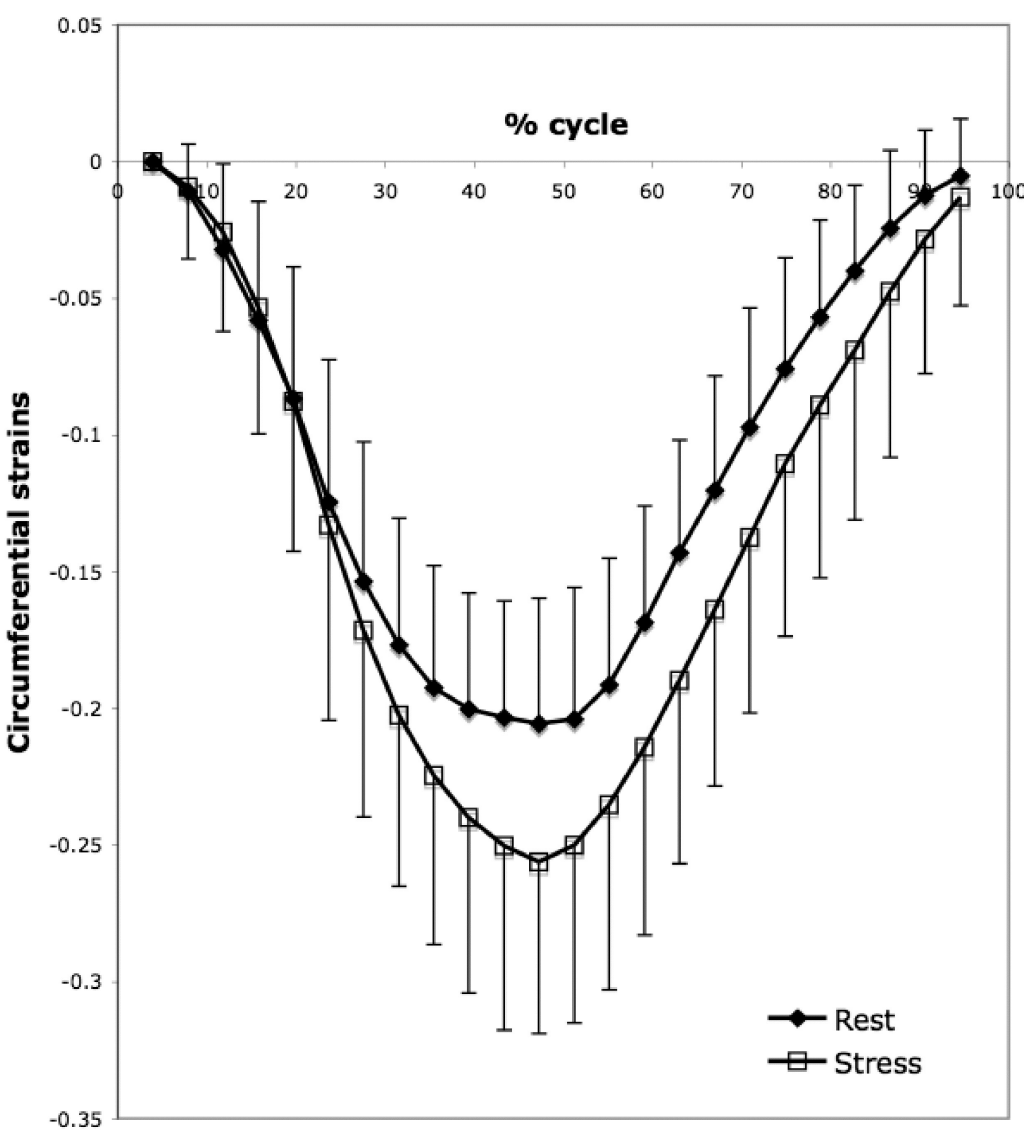
**The curves show the circumferential strain (CS) at midwall level as a function of cardiac cycle phase under rest and stress (10 μg/min/kg) obtained with tagged images.** The observed strain increase under stress in tag measurement is consistent with the increase of wall thickening measured in cine images between rest and stress. No difference in the systolic and diastolic strain (dCS/dt) rate was induced by the dobutamine.

Dobutamine induced a progressive and proportional decrease in the end systolic volume of the left ventricle (p < 0.001) without any significant changes in the end diastolic volume (p > 0.05) as presented in table 1. As a consequence, a significant increase of the LVEF (LVEF + 18.6% at 10 μg/min/kg) as well as the wall thickening (WT +88% at 10 μg/min/kg) was observed for all the dobutamine doses. In addition to a dose-dependent increase in heart rate with dobutamine (+ 19% at 10 μg/kg/min by comparison to the basal value), stroke volume also increased (+ 22%), and therefore the calculated cardiac output increased more than 45% with the highest dose (Table 1). Regarding tag imaging, the maximum circumferential strain increased significantly under the influence of dobutamine even at the low 2.5 μg/min/kg dose as shown in figure 3. However, this effect reached a maximum at 5 μg/min/kg with a 33% increase of CS by comparison to the resting value. No further CS change was observed at 10 μg/min/kg dose of dobutamine. When expressed as relative time unit (cardiac phases in % of the complete cardiac cycle), all the CS curves had the same shape with almost identical systolic or diastolic up-slope (Figure 4).

**Table 1:** Values of left ventricle end-diastolic, end-systolic volumes, stroke volume, ejection fraction and heart rate in normal rats at rest and during incremental doses of dobutamine (mean ± SD)

**Dobutamine dose (μg/min/kg)**	**0**	**2.5**	**5**	**10**
**LVEDV (μl)**	609 ± 73	604 ± 50	616 ± 67	607 ± 73
**LVESV (μl)**	158 ± 36	117 ± 29	76 ± 24 (*†)	56 ± 12 (*†)
**Stroke volume (μl)**	451 ± 54	487 ± 47	540 ± 59	551 ± 78
**Ejection fraction(%)**	74.2 ± 4.4	80.6 ± 4.6 (*)	87.7 ± 3.7 (*†)	90.7 ± 2.4 (*†)
**Heart rate (bpm)**	325 ± 31	337 ± 38	350 ± 29	386 ± 33
**Cardiac output (ml/min)**	147 ± 17	164 ± 18	189 ± 17 (*)	213 ± 26 (*)

*: p < 0.05 vs 0; †: p < 0.05 vs 2.5 μg/min/kg

According to both intra and interobserver studies, tag analysis was less variable and more reproducible than the wall thickening measurements from the cine (table 2). At rest, the interobserver variability was 19.0%, 10.0% and 13.1% in circumferential strains at epicardium, midwall and endocardium levels respectively, and 29.5% for wall thickening. The intraobserver study showed a similar trend in both cine and tag analysis. Based on cine images, ejection fraction variability was less than 3%.

**Table 2:** Inter and intra observer variabilities of wall thickening (WT) measurements and circumferential strains (Ecc) measurements at epicardium, midwall and endocardium levels

	**Inter observer variability (%)**				**Intra observer variability (%)**			
**Dose**	**0.00**	**2.50**	**5.00**	**10.00**	**0.00**	**2.50**	**5.00**	**10.00**

**Ecc epi**	19.00	20.64	24.11	21.73	14.22	12.42	14.82	19.18
**Ecc mid**	10.01	12.07	12.21	10.34	9.07	7.88	10.63	10.65
**Ecc endo**	13.50	14.67	11.90	16.51	10.57	9.56	10.86	12.31
**WT**	29.55	33.17	22.22	15.68	15.62	16.36	15.67	9.72

## Discussion

This study demonstrates that cine and tag CMR acquired on a 1.5 T clinical MR system are sensitive enough to detect the effects of a low dose dobutamine challenge on the cardiac function of normal rats. Even at the lower dobutamine dose of 2.5 μg/min/kg a positive inotropic effect can be measured by CMR in small animals.

Both cine and tag sequences allow to obtain a high image quality with high spatial and temporal resolution. Either in rest or stress conditions, regional and global cardiac function changes were well depicted by cine or tagged methods. Especially, the cine sequence had a superior spatial resolution compared to the C-SPAMM sequence, but a lower temporal resolution (inter-image 15 msec vs. 8 msec for tag sequences). According to our CMR sequence, the inter-image delay was equal to the TR. Due to the gradient limits of our MR system, it was not possible to decrease the minimum TR at the FOV needed to image rodent. According to the Nyquist-Shannon sampling theorem, a time series must be sampled at least twice as fast as its largest frequency component for an exact reconstruction. At a cardiac heart rate of 350 bpm, 6 harmonics are sampled during a cardiac cine with an inter-image delay of 15 msec. As deduced from the simulations of Setser et al [16], less than 5% error is induced on the different LVED and LVES volumes. In our setting, the number of cardiac phases was at least eleven phases per cycle for the cine CMR. This was also in accordance with a previous study performed on patients to define the minimum phase number to accurately measure the cardiac function [17]. Therefore, adequate temporal resolution for rest and stress imaging in rat was obtained for both cine and tag CMR sequence on our clinical 1.5 T MR system.

We observed a good agreement between the global function measured with CMR cine images and hemodynamic parameters. The absence of change of the LVEDV and the decrease of the LVESV are well correlated with the LV end-diastolic pressure and the peak LV pressure respectively. The contractility expressed as the dP/dt value is in accordance with the MR regional contraction measurement using wall thickness measurements from the cine CMR. The increase of wall thickening by dobutamine as measured by cine CMR was similar to previous studies performed by Nahrendorf et al [18,19]. The contractility as assessed by circumferential strains also increases significantly under stress by comparison to rest. Significant differences were observed at the subendocardium with a preservation of the strain gradient making tag CMR a suitable tool to resolve the transmural response to the low dose dobutamine. However, there was a trend for CS to saturate at the highest dobutamine dose. At 10 μg/min/kg circumferential strains reached a plateau. This saturation was previously reported in humans [20,21]. In mice also, no difference between 20 and 40 μg/min/kg dobutamine dose was found in the circumferential strain when compared to the basal state suggesting a CS saturation at dobutamine high dose [22]. One assumption given by Scott et al. [20] is the decrease of the preload at high dobutamine dose. In this case, circumferential strains could be lower than expected due to a decrease of end-diastolic volume despite an increased contractility. In this study, we observed no changes in either LVEDV or LVEDP that could support this hypothesis. The successive doses of dobutamine may also produce a desensitization of dobutamine receptors and consequently an underestimation of the dobutamine effect at the highest dose. Furthermore, since 20 to 30 minutes were necessary for each dose, the injected volume of the dobutamine solution was around 3 ml. This volume can participate to an increase of the circulating volume which can consequently limit the dobutamine effect. However, the haemodynamic studies as well as the cine studies do not suggest such effect. A possible CS underestimation at high dobutamine especially in the subendocardium region may be advocated. Due to the increase heart rate following high dose of dobutamine as well as the duration of the CSPAMM pulse by respect to the cardiac cycle, the myocardium tagging no longer occurs in end-diastole but rather in mid-systole. This induces a shift in the CS curve that can be easily corrected as previously demonstrated [12]. However, after an initial stretching during the systole, the inter-tag distance is further compressed in end-diastole limiting the efficiency of the tag line detection. Such effect may explain the apparent CS saturation observed at high dobutamine dose. A correction could consist of introducing a trigger delay to apply the CSPAMM preparation at the end of the diastole. However, such option was not used in the present study.

The intra and inter-observer analysis reveal a powerful advantage of the use of tagged images for the regional contractile function assessment in rat. Both intra and inter observer variability are around 10% for midwall circumferential strains whereas variability increased to 25% for wall thickening. Consequently, tagged images appear more suitable for regional contraction measurement as already published [23]. In particular, the low variation observed at rest or stress is promising for small change detection in stunned or hibernated myocardium or to monitor changes occurring after therapeutic interventions.

Cine CMR seems to be more affected by operator. The main difficulty in wall thickening measurement remains the myocardium segmentation. Endocardial and epicardial boundaries were manually drawn since no accurate automatic tools are available. The absence of contrast between myocardium and thoracic muscle increases the difficulty to well delineate epicardium. Since wall thickening is dependant of four contour drawings, an error of only one contour will directly be reported on wall thickening measurement. Cine images are however suitable for a global function evaluation. Due to a high contrast to noise ratio between left ventricle cavity and myocardium, endocardium segmentation is easily performed and left ventricle end systolic and end diastolic volumes can be accurately assessed. The results of ejection fraction variability (less than 3%) which is consistent with previous published results in rat [6] confirm this assumption.

### Study limitations

The modified Simpson's method was used to measure the left ventricle end systolic and end diastolic volumes instead of a summation of continuous short axis slices covering the entire left ventricle. This choice resulted from the time constraint induced by the dobutamine challenge. The modified Simpson's method requires the acquisition of only 3 slices instead of 5 to 7 slices needed to cover the entire ventricle resulting in a significant time savingThe modified Simpson's method yields a small underestimation of the left ventricle volumes without significant difference in the ejection fraction estimation [14]. Therefore, we considered reasonable to use this tradeoff as it is usually performed in the clinical dobutamine stress tests in patients. Finally, hemodynamics were obtained in open-chested preparations as opposed to the less invasive alternative using a Millar ultraminiature catheter pressure transducer [24] that would have more closely paralleled the CMR studies. The effect of the additional stress induced by the open-chest surgery on the dobutamine challenge can not be assessed by our protocol.

## Conclusion

In conclusion, cardiac cine and tagging CMR with dobutamine stress are feasible in rat at 1.5 T on a clinical system. Both sequences offer consistent measurements of regional cardiac function by comparison to a haemodynamic study. Significant changes were observed with incremental doses, even at the lowest dose tested (2.5 μg/min/kg). A 18% increase of the EF was observed with the cine CMR for a global function evaluation at high dobutamine dose whereas the increase was 33% for the tagging method in regional contractile function assessment. In addition, tag CMR permits a sub-endocardial examination and appears to be an excellent tool to quantify accurately and rapidly the heart function with a low observer variability that could particularly valuable to monitor pharmacological interventions.

## Competing interests

The authors declare that they have no competing interests.

## Authors' contributions

JLD carried out the design of the study, MR acquisition and analysis, wrote manuscript

JPJ developed the tag analysis software. JNH participated in the MR acquisition, sequence development and results discussion. PC participated in the design of the study and helped to draft the manuscript. KMA carried out the animal sacrifice and post mortem analysis. SR and DB participated in the intra and inter observer study. MLC participated in the MR acquisition and in the intra and inter observer study. DM carried out the hemodynamic study

JPV carried out the design of the study and its coordination, participated in manuscript writing. All authors read and approved the final manuscript.
